# The Power of Exercise: Buffering the Effect of Chronic Stress on Telomere Length

**DOI:** 10.1371/journal.pone.0010837

**Published:** 2010-05-26

**Authors:** Eli Puterman, Jue Lin, Elizabeth Blackburn, Aoife O'Donovan, Nancy Adler, Elissa Epel

**Affiliations:** 1 Department of Psychiatry, University of California San Francisco, San Francisco, California, United States of America; 2 Department of Biochemistry and Biophysics, University of California San Francisco, San Francisco, California, United States of America; 3 Veterans Affairs Medical Center, San Francisco, California, United States of America; University of Valencia, Spain

## Abstract

**Background:**

Chronic psychological stress is associated with detrimental effects on physical health, and may operate in part through accelerated cell aging, as indexed by shorter telomeres at the ends of chromosomes. However, not all people under stress have distinctly short telomeres, and we examined whether exercise can serve a stress-buffering function. We predicted that chronic stress would be related to short telomere length (TL) in sedentary individuals, whereas in those who exercise, stress would not have measurable effects on telomere shortening.

**Methodology and Principal Findings:**

63 healthy post-menopausal women underwent a fasting morning blood draw for whole blood TL analysis by a quantitative polymerase chain reaction method. Participants completed the Perceived Stress Scale (Cohen et al., 1983), and for three successive days reported daily minutes of vigorous activity. Participants were categorized into two groups-sedentary and active (those getting Centers for Disease Control-recommended daily amount of activity). The likelihood of having short versus long telomeres was calculated as a function of stress and exercise group, covarying age, BMI and education. Logistic regression analyses revealed a significant moderating effect of exercise. As predicted, among non-exercisers a one unit increase in the Perceived Stress Scale was related to a 15-fold increase in the odds of having short telomeres (p<.05), whereas in exercisers, perceived stress appears to be unrelated to TL (B = −.59, SE = .78, p = .45).

**Discussion:**

Vigorous physical activity appears to protect those experiencing high stress by buffering its relationship with TL. We propose pathways through which physical activity acts to buffer stress effects.

## Introduction

Chronic psychological stress can have a detrimental impact on human physical health[1], [2]. Unemployment[3], financial strain[4], caregiving for a chronically ill loved one[5], and other stressful life experiences[6] have been related to increased risks for cardiovascular disease, insulin resistance, and other markers of disease. In the past half-decade, accumulating evidence suggests that one of the pathways through which chronic stress may impact health is through accelerated cell aging, as indexed by the length of the telomeric DNA at the end of chromosomes[7]–[11]. Telomeres are DNA-protein complexes that cap chromosomal ends, promoting chromosomal stability. At each cellular division, telomeric DNA terminal regions are not fully replicated, which, if not counteracted by elongation by telomerase, can lead to telomere shortening. If telomeres shorten to a critical length, cells cease to multiply, often by entering a state of senescence characterized by, for example, increased cellular secretion of proinflammatory cytokines and loss of antigen recognition in the case of immune system cells[12]. As a result, telomere length (TL) has emerged as a widely recognized biomarker of immune cell biological ‘age’[13]. Short TL in white blood cells has been linked to a range of health problems, including coronary heart disease, and diabetes mellitus, and to early mortality[14]–[17].

In the first study to link psychological stress with short TL, it was demonstrated that among pre-menopausal women caregiving for a chronically ill child, duration of caregiving was associated with short TL[7]. Moreover, the results were not simply a result of caregiving, because in addition, greater perceived stress was associated with shorter TL consistently across the entire sample, which included control mothers of ‘healthy’ children. Yet, not all people under chronic stress develop health problems, and likely, do not have distinctly short telomeres, although no studies have yet identified potential moderators of this relationship.

Several studies have examined health behaviors as potential mediators through which stress affects health, but few have examined their potential to ameliorate the effects of stress on physiology. Here we propose that a specific health behavior, physical activity, can moderate the impact of stress on cell aging. Physical activity is related to longer telomeres, both in a national sample of twins[18], and in endurance trained athletes compared to sedentary controls[19]. Therefore, we hypothesized that stressed yet physically active adults will be more protected from the impact of stress on their TL compared to those stressed and not active. Specifically, we predicted that chronic stress will be related to short telomeres only in those inactive (defined as not engaging in recommended amounts of physical activity across a three day period) while physically active adults will show no association between stress and TL. We examined these associations and the protective capacity of exercise in 63 post-menopausal healthy, non-smoking women.

## Results

Table 1 presents means and standard deviations for perceived stress, physical activity, and TL and covariates. Participants with higher perceived stress were significantly less likely to exercise (*r* (61) = −.27, p = .03), had shorter TL (*r* (57) = −.29, p = .03), higher BMI (*r* (61)  = .26, p = .04) and fewer years of education (*r* (61)  = −.27,  = .03). Across the sample as a whole, minutes of physical activity and TL were unrelated to each other (*r* (58)  = .04, *p* = .74), and neither was related to any of the potential covariates described above.

**Table 1:** Descriptives across sample (means and standard deviations).

	Mean	SD
**Perceived stress (item mean)**	1.55	.77
**Physical activity (minutes over 3 days)**	45.4	64.3
**Telomere length (base pairs)**	5502.73	700.82
**Age (years)**	61.87	6.51
**BMI (kg/m^2^)**	26.57	5.41

**Figure 1:**
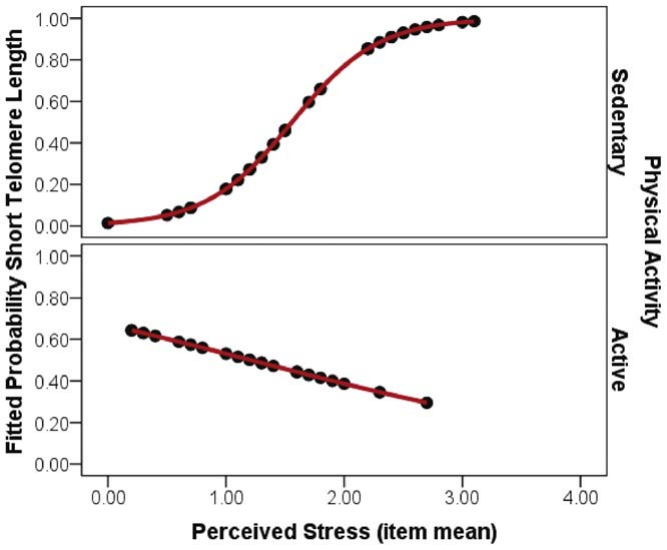
Fitted Probability of short telomeres as a function of perceived stress for sedentary and active individuals. Note. Physical activity categories are based on whether the participant met CDC recommended levels of exercise per week. Perceived stress ratings are based on the Perceived Stress Scale. The interaction effect was significant (p<.05), indicating that the relationship between perceived stress and telomere length was significant in inactive participants only. The Y axis probability presents the probability of categorization into short telomere length (bottom tertile) as a function of perceived stress in inactive (top of figure) versus active (bottom of figure) participants. Probability scores were calculated from the fitted regression equations, assuming mean BMI and education level.

**Figure 2:**
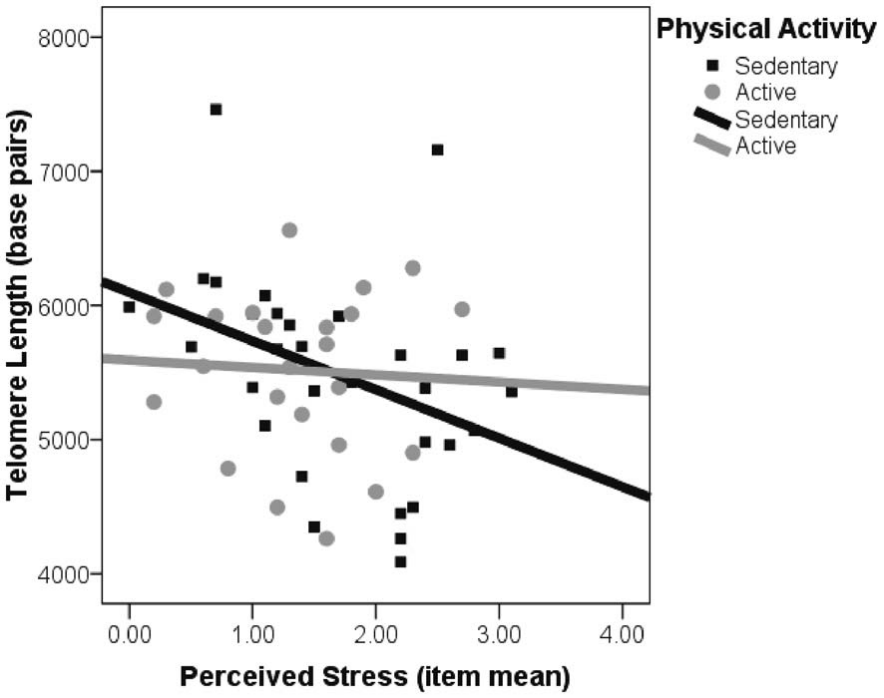
Relationship between perceived stress and telomere length as a function of physical activity. Note. Physical activity categories are based on whether the participant met CDC recommended levels of exercise per week. Perceived stress ratings are based on the Perceived Stress Scale. The relationship between perceived stress and telomere length was significant in sedentary participants only.

Next, we examined whether physical activity (1 = meets or exceeds recommended amount, active vs. 0 = does not meet recommendation, sedentary) moderated the stress-TL relationship. In a logistic multiple regression analysis, we compared a dichotomous telomere outcome, the two extreme TL groups: those in the highest (n = 20) and lowest (n = 21) tertiles of TL. We predicted the likelihood of having short telomeres as a function of the direct effects of stress and exercise group, and the interaction effect of the two groups as well. Covarying education, and BMI, the likelihood of having short telomeres was greater with higher levels of perceived stress (p<.05), however this relationship was significantly moderated by exercise category (interaction b = −3.32, SE = 1.40, p = .02). Given that age[20], [21] and antioxidant use[22] have been associated with TL in past studies, we repeated all analyses including these two variables as covariates. Results were similar even after including age and antioxidant vitamin intake (interaction b = −3.34, SE = 1.38, p = .02). For those who exercised less than the recommended amount, a one unit increase in the perceived stress scale mean item response was related to a 15-fold increase in the odds of having short telomeres (B_0_ = −4.27, B_stress_ = 2.74, SE_stress_ = 1.09, OR = 15.48, p = .01), controlling for mean-centered education and BMI. On the other hand, perceived stress appeared to have little effect on telomere category (short versus long) in those participants who exercised the recommended amount over a three day period (B_0_ = .71, B_stress_ = −.59, SE_stress_ = .78, OR = .56, p = .45), controlling for mean-centered education and BMI. Figure 1 illustrates the fitted probability (from 0 to 1) of being categorized as having short telomeres as a function of perceived stress for inactive versus active participants. Probability scores were fitted from each model from the logistic regression equation, at mean BMI and education level. Further, for illustrative purposes, we present a scatter-plot graph (Figure 2) that predicts the entire sample of TL as a function of perceived stress for active and inactive participants. As seen in Figure 2, higher levels of perceived stress significantly predict decreased TL in inactive participants only.

As a secondary analysis, to more sensitively test the level of exercise that matters most, we employed the Johnson-Neyman statistical approach[23] to examine the significant confidence intervals at which reported minutes of physical activity protected participants from the stress effects on TL. At values of exercise below 42 minutes across the three days, perceived stress was significantly related to the odds of having short TL. On the other hand, at values higher than 42 minutes, perceived stress was unrelated to TL (see Table 2). Thus, at least 14 minutes a day of vigorous exercise appears to be a critical amount for protection from the effects of stress.

**Table 2:** Johnson-Neyman significance regions for perceived stress predicting telomere length at values of physical activity minutes.

Number of minutes of physical activity	Log-odds for perceived stress	SE	Confidence interval (LLCI,ULCI)
0	2.31	.94	.45,4.17
10	2.04	.84	.40,3.68
21	1.76	.74	.32,3.22
32	1.50	.67	.19,2.80
**42**	**1.22**	**.62**	**.00, 2.42**
53	.95	.62	−.24, 2.15

The Johnson-Neyman technique permits the examination of the log-odds of having short telomeres as a function of perceived stress at different values of physical activity (defined by statistical software). Confidence intervals that do not pass 0 are considered significant. As can be seen in bold, at 42 minutes of vigorous physical activity over 3 days, stress no longer significantly predicted telomere length. SE = standard error; LLCI and ULCI = lower and upper limit 95% confidence intervals, respectively.

Lastly, recent evidence suggests that female estrogen-deficient mice express reduced telomerase activity, and that hormone replacement (HR) restores the levels to those of control mice.[24] In light of these findings, we reran the above analyses removing the two participants in our sample who were undergoing HR therapy. Results were similar with and without these participants and thus results presented contain all participants.

## Discussion

Consistent with previous studies[7], [9], [10], our data indicate that higher perceived psychological stress is associated with shorter TL. Importantly, physical activity was able to protect those experiencing stress by buffering its relationship with TL, as hypothesized. Our findings support the physical benefits of activity, and, interestingly, map well onto Centers for Disease Control (CDC) recommendations for amount of physical activity required to sustain a healthy body (75 minutes of vigorous exercise per week). Furthermore, physical activity buffered the impact of stress on TL even after covarying for factors clearly associated with stress, physical activity or telomere—education[25], age[13], [21], body mass index[17], and antioxidant vitamin use[23], [26]. For example, lower socioeconomic status individuals are more likely to experience stress[25] and engage in less leisure time physical activity[27]. Physically unfit and stressed individuals are often more at risk for obesity and other health problems[28], [29]. In the present study, we were able to rule out these potential factors as alternative explanations for both the stress-telomere association and the buffering potential of physical activity.

The mechanisms by which stress impacts the body at the cellular level are unknown, but one likely pathway is telomerase activity. Telomerase is a cellular ribonucleoprotein reverse transcriptase enzyme that adds telomeric DNA to shortened telomeres, and protects them[30]. Chronically stressed individuals have lower telomerase activity[7] and often demonstrate altered glucocorticoid profiles[1]. Higher excretion of urinary stress hormones is cross sectionally related to lower telomerase and shorter telomeres[8], [9]. Further, a recent experimental study demonstrated that exposing human lymphocytes, in vitro, to high levels of cortisol during primary stimulation and upon subsequent re-stimulation can dampen telomerase activity[31]. Autonomic nervous system profiles associated with chronic stress (i.e. high blood pressure, lower heart rate variability) are also associated with lower telomerase activity[8].

Physical activity training augments telomerase activity in myocytes and circulating mononuclear cells in rats[19], [32], and in human leukocytes [19], thus providing a plausible and potentially testable pathway to explain our findings. Exercise might also buffer telomere shortening through affecting the balance between oxidative stress and antioxidants. Chronic stress has been linked to increased oxidative stress[7], [33], and is known to shorten telomeres and inhibit telomerase activity in cells *in vitro* [34], [35]. Aerobic training interventions can enhance endogenous antioxidant activity in human and animal models[36], [37], although not all studies on this have shown consistent findings[38]. While vigorous physical activity is paradoxically linked to increased oxidative damage[39], exercisers nevertheless have lengthened telomeres[19]. Recent models of exercise propose that physical activity promotes a ‘eustress’ state[40], whereby exercise, at moderate levels, promotes manageable and beneficial effects[41]. On the other hand, extreme exercise promotes catabolic processes and ultimate cell damage rather than strengthening [42].

Furthermore, physical activity may act to protect highly stressed individuals through autonomic, neuroendocrine and cognitive pathways. Sympathetic nervous system and cortisol activation in response to stress is often blunted in physically fit humans and other animals[43]–[45]. Heart rate variability is improved in response to fitness training[46]. Cognitive responses to stress, such as rumination, are associated with enhanced cortisol responses to stress[47]. Exercise induction, which leads to decreased rumination across time and enhanced feelings of self-efficacy[48], may reduce the cognitive processing thought to activate neuroendocrine and autonomic responses to stress.

In the present study we also examined the interval of physical activity above which stress no longer significantly predicts TL. The data indicated that above around 40 minutes of vigorous activity over 3 days, stress is no longer associated with short telomeres. Forty minutes over 3 days correspond well with the CDC-recommended levels of vigorous activity, as well as those recommended by others for healthy living[49], [50]. An assumption in the present study was that a 3-day accumulation of reported physical activity represented our participants' typical engagement of exercise, and a long-term lifestyle factor. Future studies should be designed to measure participants' baseline fitness as well as daily reported engagement in activity for longer durations. These studies could better delineate the number of weekly minutes of exercise required to protect stressed individuals, and to observe actual benefits of physical activity at the cellular level.

The findings of the present study underscore an inherent problem in promoting physical activity in stressed individuals: while physical activity may confer more benefits to highly stressed individuals, they are simply less likely to actually exercise. It will be helpful to better understand the role of contextual factors (such as personality, motivation, and daily events) that promote physical activity in stressed versus non-stressed individuals. In contrast to previous studies on physically fit adults and telomeres[18], [32], we did not demonstrate direct associations between physical activity and TL across our entire study sample. Work by Cherkas and colleagues[18] demonstrating a relationship between physical exercise and longer TL used a larger sample of adults (*N*>2500) with a wider age range of participants and physical activity categories. Our smaller sample size, restricted age range, and definition of physical activity may explain our divergent finding, although further examination of this relationship with a larger sample size is required.

In summary, the novel findings reported here support the buffering potential of physical activity on the detrimental effects of stress on cellular longevity. While we ruled out several lifestyle factors previously linked to TL (e.g., BMI, vitamin intake), the possibility remains that other health promoting behaviors may also have similar buffering effects, or that other factors associated with both exercise and TL may be at work. Intervention studies are still needed to provide a better test of this relationship. Intervention studies have already shown that exercise can improve major depression[51], which is another sequela of chronic stress[1], [52]. It is reasonable to conclude that exercise should be strongly advised and prescribed to people reporting high levels of psychological stress.

## Materials and Methods

### Participants and procedure

Sixty-three healthy post-menopausal women aged between 54 and 82 years were recruited through flyers and posters in the community, and from service providers serving the elderly in the San Francisco Bay Area. They were part of a prospective study on caregiving and its effects on physical and psychological well being. Women were recruited specifically for having varying levels of stress levels, and so included both dementia caregivers and non-caregivers, each showing a range of stress levels but with higher levels in the caregivers. The sample described here included 50 White (79.7%), three Black, one Hispanic/Latina, six Asian women, and two women of Asian and White background. Seventy percent of participants had at least a Bachelor's degree, and the average household income fell between $60,000 and $69,000. Exclusion criteria included the presence of major medical conditions such as heart disease, cancer, or diabetes, use of medications containing agents known to affect stress hormone levels, and regular smoking. In a fasted state, participants had a catheter insertion, and after resting for an hour, they underwent a blood draw between 8.00 AM and 10.00 AM, from the non-dominant arm. Participants completed self-report questionnaires and exercise logs within one week of the blood draw. The study protocol was approved by the Institutional Review Board of the University of California, San Francisco. Written, informed consent was obtained from all participants.

### Materials and measures

#### Perceived stress

The 10-item Perceived Stress Scale[53] was used to assess appraisals of psychological stress experienced during the last month, including the extent to which situations are experienced as unpredictable, uncontrollable and overwhelming. Participants are asked to rate the extent to which they felt or thought a particular way in the previous month on a 5-point Likert scale ranging from 0 (“never”) to 4 (“very often”). Internal consistency was high (a = .93).

#### Physical Activity

Participants reported on a daily basis for three successive days their amount of vigorous exercise, defined as “increased heart rate and/or sweating.” Higher levels of self-reported physical activity are associated with greater fitness, as measured by the metabolic equivalent (MET)[54]. Retrospective reporting is plagued by recall bias[55], and thus daily reporting of behaviors is considered a stronger measure of behavior[56], [57]. Daily reports of physical activity were extremely skewed (range 0 to 300 minutes, 30 participants reported no exercise), and therefore we split participants based on previously reported required amounts of physical activity for good health. The Center for Disease Control and Prevention[58], based on previous work[59], recommends an average of 75 minutes of vigorous activity per week (an average of 33 minutes for a three day period). We thus split participants based on meeting these recommended guidelines (less than 33 minutes = 0, equal or greater than 33 minutes = 1), labeled here, for brevity, as “sedentary” vs. “active”. We also report physical activity as a continuous measure for our means, standards deviations, correlations and t-tests.

#### Telomere length (TL)

Samples were collected in 10-ml heparin tubes (Becton–Dickinson, Franklin Lakes, NJ). Leukocytes were isolated and frozen at −80°C. DNA was extracted from leukocytes by the University of California San Francisco DNA bank. Genomic DNA isolation was performed using a standardized and quality-controlled PureGene DNA isolation system (Gentra Systems, Minneapolis). The quantity and quality of the genomic DNA isolate was determined by 260/280 UV spectrophotometery. At regular intervals, the integrity of isolated DNA was evaluated by agarose gel electrophoresis performed on randomly selected isolates.

DNA was analyzed for TL using quantitative polymerase chain reaction (qPCR) as previously described[60] with the following modifications. The primers for the telomere PCR were *tel1b* [5′-CGGTTT(GTTTGG)_5_GTT-3′], used at a final concentration of 100 nM, and *tel2b* [5′-GGCTTG(CCTTAC)_5_CCT-3′], used at a final concentration of 900 nM. The primers for the single-copy gene (human beta-globin) PCR were *hbg1* [5′ GCTTCTGACACAACTGTGTTCACTAGC-3′], used at a final concentration of 300 nM, and *hbg2* [5′-CACCAACTTCATCCACGTTCACC-3′], used at a final concentration of 700 nM. Serial dilutions of genomic DNA from the Hela cancer cell line were used as the reference DNA to create the standard curve. The quantities of telomeric product (T) and single copy gene (S) were determined relative to the reference DNA by the standard curve method. All PCRs were carried out on a Roche Lightcycler 480 real-time PCR machine with 384-tube capacity (Roche Diagnostics Corporation, Indianapolis, IN). The telomere thermal cycling profile consists of: cycling for T (telomeric) PCR: denature at 96°C for 1 second, anneal/extend at 54°C for 60 seconds, with fluorescence data collection, 30 cycles; cycling for S (single copy gene) PCR: denature at 95°C for 15 seconds, anneal at 58°C for 1 second, extend at 72°C for 20 seconds, 8 cycles; followed by denature at 96°C for 1 second, anneal at 58°C for 1 second, extend at 72°C for 20 seconds, hold at 83°C for 5 seconds with data collection, 35 cycles. Details of the TL measurement method are described elsewhere[61].

#### Covariates

Covariates examined were relevant factors known to be associated with TL in previous studies. In the present study we examined age in years, education (1 = less than 12 years, 2 = high school graduate, 3 = some college or technical school, 4 = AA degree, 5 = bachelor's degree, 6 = advanced degree), anti-oxidant vitamin intake (binary coded; 48 reported taking anti-oxidant vitamins and 15 reported no intake) and body mass index (BMI). BMI was calculated as weight in kilograms (measured on a balance beam scale in hospital gown) divided by height in meters squared. To maintain power, we retained only the covariates that were related to any of our predictors or outcome.
